# Examining the correlates of meal skipping in Australian young adults

**DOI:** 10.1186/s12937-019-0451-5

**Published:** 2019-04-03

**Authors:** Felicity J. Pendergast, Katherine M. Livingstone, Anthony Worsley, Sarah A. McNaughton

**Affiliations:** Institute for Physical Activity and Nutrition (IPAN), School of Exercise and Nutrition Sciences, Deakin University, Geelong, Australia

**Keywords:** Meal skipping, Young adults, Correlates, Eating patterns

## Abstract

**Background:**

Meal skipping is associated with diet-related chronic disease risk and is highly prevalent in young adults. Despite this, the correlates of meal skipping in this population group are unknown. Therefore, the aim of this study was to examine the prevalence and correlates of meal skipping in young adults.

**Methods:**

Young adults aged 18–30 years (*n* = 578) (24% male, 76% female) used ‘FoodNow’, a purpose designed real-time smartphone application to record food and beverage consumption over four non-consecutive days. The day following each reporting day, participants were asked about their previous day’s eating occasions; if any eating occasions were not reported or if any were skipped. These data were used to categorise participants into specific meal skippers (breakfast, lunch and/or dinner skipper). Participants also completed an online questionnaire, which contained measures of correlates from the social-ecological framework across the individual, social-environmental and physical-environment domains. Logistic regression analyses were used to examine associations between specific meal skipping behaviours and measured correlates.

**Results:**

Individual domain correlates (education status, smoking status and time scarcity) were associated with varying meal skipping behaviours, while no correlates from the social-environmental or physical-environmental domains of the social-ecological framework were associated with any meal skipping behaviours. Participants with a university education were less likely to be a meal skipper (any meal) (OR = 0.46; 95%CI: 0.22, 0.95; *p* = 0.035), while those who previously or currently smoked cigarettes were more likely to be breakfast skippers (OR = 1.10; 95%CI: 1.15, 3.86; *p* = 0.016) compared to those who had never smoked before. Those who are time scarce were more likely to be either breakfast (OR = 1.12; 95%CI: 1.00, 1.26; *p* = 0.036) or lunch skippers (OR = 1.11; 95% CI: 1.01, 1.23; *p* = 0.033). No variables were significantly associated with dinner skipping.

**Conclusions:**

The findings suggest that the correlates of meal skipping vary according to the specific meal skipped. University education status needs to be considered when designing interventions aimed at the reduction of meal skipping among young adults, while correlates such as time management and smoking status may offer potential behaviour change targets within these interventions.

**Electronic supplementary material:**

The online version of this article (10.1186/s12937-019-0451-5) contains supplementary material, which is available to authorized users.

## Background

Frequently skipping meals, particularly the breakfast meal, is associated with a number of nutrition related outcomes. These include poor diet quality [1], lower intakes of vitamins and minerals [2], higher total energy intake [1], and chronic disease risk factors such as central adiposity [3, 4], markers of insulin resistance [4] and cardio metabolic risk factors [4].

Young adults, defined as those aged 18–30 years [5] have some of the highest rates of meal skipping when compared with other age groups [6]. Data from the American National Health and Nutrition Examination Survey (NHANES) 2011–12 suggests that 33% of young adults (19–29 years) do not consume breakfast on any given day [7]. While, findings from the nationally representative Australian Health Survey 2011/12 showed that 39% of Australian young adults (19–24 years) ate breakfast less than 5 days per week, compared with 10% of children (8–11 years) and 4% of older adults (> 65 years) [6]. While much of the research is focused on breakfast skipping exclusively, a recent systematic review reported that meal skipping (any meal) rates in young adults (18–30 years) ranged between 5 and 83%, with rates for skipping specific main meals varying: breakfast (14–89%), lunch (8–57%), dinner (5–47%) [8]. Despite the health implications of meal skipping and its increased prevalence among young adult populations, limited research has explored the correlates of this eating behaviour.

A recent systematic review of 35 studies, identified 20 unique correlates which have been previously assessed in relation to meal skipping [8]. Sex was the most frequently assessed correlate of meal skipping, with males being more likely to skip breakfast, and females being more likely to skip the lunch or dinner meal [9–11]. The concept of time or the lack of time has also been frequently assessed as one of the main self-reported influences on meal skipping [12–14]. Correlates of meal skipping may also differ depending on whether the meal skipped is breakfast, lunch or dinner. For example, research exploring meal skipping in university students in Ghana reported breakfast skipping to be influenced by a lack of time and lack of hunger, compared to dinner skipping which was influenced by busy schedules and weight watching [15]. Many of the studies within this review did not assess correlates related to specific meal skipping behaviours (breakfast, lunch or dinner), thus were limited to assessing correlates related to total meal skipping [8].

Conceptually, the definition of meal skipping is the omission or failure to consume one or more traditional main meal (breakfast, lunch or dinner) throughout the day [16]. However, the current methods used to measure and define meal skipping within the literature are diverse and provide data limited by recall bias, single reporting days, and may not be capable of determining specific meal skipping behaviours [8]. Dietary intake assessments such as 24-h recalls, and food diaries have been used previously, yet have required adaptations to measure meal skipping and their correlates [11, 17]. These methods are limited by participant recall bias and can result in a high participant and researcher burden due to lengthy interviews or data coding processes [18]. Of the studies which used these methods to measure meal skipping, single days of dietary data were analysed which may not be optimal when trying to examine usual behaviours of meal skipping and its correlates [11, 17]. Specially designed food frequency questionnaires have also been used previously [19], yet are often limited in their ability to provide information on what influences specific meal skipping occasions as they are designed to report averages or usual intakes over a substantial time frame (months or even a year) [18]. Binary response questions, numerical response items and multiple-choice items have also been used within the literature [10, 20, 21], making comparisons of studies difficult due to the variety in response options.

In conclusion, there have been few studies of the correlates of the skipping of meals in young adults and little examination of correlates associated with skipping particular meal types [8]. The identification of specific meal correlates may inform the design of more effective and targeted public health messages aimed at limiting meal skipping in young adult populations. Therefore, the aim of this research was to examine the prevalence and correlates of skipping any meal and, specifically, breakfast, lunch and dinner in a young adult population using a validated real time assessment method.

## Methods

Data for this study were collected as part of the Measuring EAting in everyday Life Study (MEALS), a cross-sectional study of young adult participants conducted between April 2015 and April 2016. Participants were aged between 18 and 30 years inclusive, living in Victoria, Australia. Women who were currently pregnant or lactating were not recruited because of potential variations in their eating habits and food intake. Recruitment was conducted using social media (Facebook, Twitter) and paper-based methods (poster advertisements, flyers). After providing written consent, participants completed an online questionnaire (via Qualtrics, an online questionnaire hosting platform) and the FoodNow food diary application (app). Following completion of the study, a thank you letter and a $25 voucher were sent to each participant. Ethical approval was granted by the Deakin University Human Ethics Advisory Group, Faculty of Health in February 2015 (HEAG-H 11_2015).

### Measures

#### Meal skipping

Meal skipping was assessed using data collected from the FoodNow app. Participants were asked to record all eating and drinking occasions in the FoodNow app on four non-consecutive days (three weekdays and one weekend day) over an eight day period [18]. Reminders/prompts to use the FoodNow app were sent via ‘push’ notifications if nothing was reported in the app for a three-hour period during waking hours (9 AM-9 PM). Participants also completed questions on the day following each active reporting day. These “following day” questions included items relevant to the previous day’s sleep times, supplement use, meal skipping and misreporting behaviours. The question used to assess meal skipping was *“Were there any eating occasions that you did not eat/skip?”* Response options were *yes/no*. If *yes* was selected the name of the eating occasion/s that were skipped/not eaten were selected from a drop-down menu. Misreporting was measured by *“Were there any eating occasions that you did not report?”* Response options were *yes/no*. If *yes* was selected the name of the eating occasion/s that were not reported were selected from a drop-down menu. These following day questions aimed to distinguish between eating occasions that were skipped on the reporting day versus eating occasions that were eaten but not reported (i.e. reporting omissions).

An evaluation of the FoodNow food diary app comparing energy intake and energy expenditure assessed using SenseWear Armband (BodyMedia Inc., USA), has shown good agreement between these methods [22], with further details of the FoodNow app available elsewhere [22].

Participants were categorised into meal skippers (any meal), breakfast skippers, lunch skippers and dinner skippers based on the following criteria. Meal skipping (any meal) was defined as skipping ≥25% of all main meals (breakfast, lunch and dinner) across their reporting days [3]. Therefore, across four days of reporting, there were 12 possible main meals, with a meal skipper (any meal) identified as those skipping four or more traditional meals. Specific meal skippers were categorised with a similar criterion; skipping ≥ 25% of the specific meal across the reporting period [3]. An individual participant could be categorised as a meal skipper (any meal) and/or a specific meal(s) skipper (i.e. they could be both a breakfast and lunch skipper). This definition was based on previous research in this field [3].

#### Correlates of meal skipping

Potential correlates of meal skipping were measured using the online questionnaire which included measures from three domains of the social-ecological framework (individual, social-environmental and physical-environment) as originally proposed by Story et al. [23]. The social-ecological framework has previously been used in nutrition [23] and combines ecological perspectives with social cognitive theories resulting in a multi-level framework that is useful for understanding and examining correlates of eating behaviours. Potential influences from the macro system domain of the social-ecological framework were not assessed within this study, due to their distal and indirect role in influencing meal skipping [24]. Where possible, previously developed items from the literature with known reliability were used to measure correlates of meal skipping. Additional file 1: Table S1 presents the full list of measures included in the questionnaire with response options and data on reliability.

#### Individual level correlates

Demographic variables were measured in the online questionnaire. Education was categorised as “holding a university degree” or “not having a university degree” [25]. Ethnicity was measured as country of birth and was categorised as “born in Australia” and “born outside of Australia”. Participants were also asked if they avoided any foods due to culture, religion or ethical reasons (yes or no) [26].

Smoking status was categorised as “never smoked”, or “ex, occasionally or regular smoker” [25]. These cuts points were chosen as previous research suggests that ex-smokers retain dietary behaviours similar to those of current smokers [27]. Physical activity (PA) was measured by the International Physical Activity Questionnaire (IPAQ) - Short form [28]. This validated questionnaire assesses PA, specifically walking, moderate-intensity activities and vigorous intensity activities in both frequency and duration. From these items total minutes/week of various intensities of PA were calculated. Time spent in vigorous and moderate intensity PA per week was compared with the national guidelines for PA (> 150 min of moderate or > 75 min of vigorous activity per week) [29] to categorise participants into whether they “met” or “did not meet PA guidelines”.

A range of pre-existing validated multi-item scale measures were included in the online questionnaire to measure time scarcity, self-efficacy, food insecurity and general nutrition knowledge. Time scarcity was measured by a four item measure used previously in Project EAT [30], with items scored 1–4 forming a composite score 4–16. Self-efficacy was measured by an adapted version of Sallis’s self-efficacy scale for health-related diet and exercise behaviours [31]. The 17 items were summed resulting in scores between 17 and 85. Food insecurity was measured by the guide to measuring household food security scale [32], which is a five-item scale with each affirmative response awarded one point. The responses were summed to form a scale with a score ranging from 1 to 5. The 51 item General Nutrition Knowledge Questionnaire was used to assess participants’ nutritional knowledge, with each correct item awarded one point. These were then summed to form a score from 0 to 51 [33].

Mood, hunger, weight control, habit and taste were assessed using items which asked participants “*How likely are the following factors going to result in SKIPPING a main meal (breakfast, lunch or dinner)?”* Five-point Likert response scales were used (not at all likely to extremely likely). Due to lack of existing measures, these items were created specifically for this study. Test-retest reliability was examined in a separate sample of young adults (*n* = 90) with items administered two weeks apart, with agreement measured by weighted kappas. Moderate levels of agreement (weight kappas 0.41–0.60) were recorded for the mood, hunger and habit measures, with fair agreement (weight kappas 0.21–0.40) for weight control and taste.

#### Social-environmental correlates

The social-environmental correlates included relationship status and the preferences of others at the eating occasion. The preferences of others were measured by items that asked participants “*How likely are the following factors going to result in SKIPPING a main meal (breakfast, lunch or dinner)?”* These items were assessed on five-point Likert scales (not at all likely to extremely likely). Test-retest results found fair agreement (weight kappas 0.21–0.40) for this scale. Relationship status was measured in the online questionnaire and was categorised as “in a relationship” or “not in a relationship”.

#### Physical-environment correlates

Housing type and socio-economic living environment [34] were investigated for associations with meal skipping. Current housing or living situation was aggregated into three categories based on living with family, friends or alone. Social-economic position was measured using the Socio-economic Index for Areas (SEIFA) Index for Relative Socio-economic Disadvantage [34]. Postcodes were used to assign a composite index score. These scores were derived from Census data relating to attributes such as low income, low educational attainment and unskilled occupations and were categorised into high, medium or low categories.

#### Covariates

Body mass index (BMI), sex and age were considered as covariates in this study, as recent literature suggests that these variables are associated with meal skipping and the included correlates [8, 15]. Height (cm) and weight (kilograms) were self-reported by participants in the online questionnaire and BMI was calculated as weight (in kilograms)/height (in metres^2^) [35].

### Data analysis

Logistic regression analyses were used to examine associations between potential correlates (dependant variable) and meal skipping (any meal), breakfast skipping, lunch skipping and dinner skipping (independent variable). Bivariate logistic regression analyses (Model 1) were used to examine associations between each potential correlate and each meal skipping behaviour (e.g. meal skipping (any meal)). Only variables that were statistically significant in model 1 were included in model 2 (multivariate logistic regression analyses). All variables included in model 2 were tested for multicollinearity using tolerance and variance inflation factors (VIFS); no multicollinearity was identified. All models were adjusted for age (continuous), sex and BMI (continuous). Results are reported as odds ratios (OR) and considered statistically significant at a *P*-value of < 0.05. STATA version 14 (Stata Corporation) was used for all analyses.

STROBE-nut was reference throughout the preparation and conduction of this study to help strengthen the delivery and reporting of observational nutritional studies (Additional file 2: Table S2) [36].

## Results

**Fig. 1:**
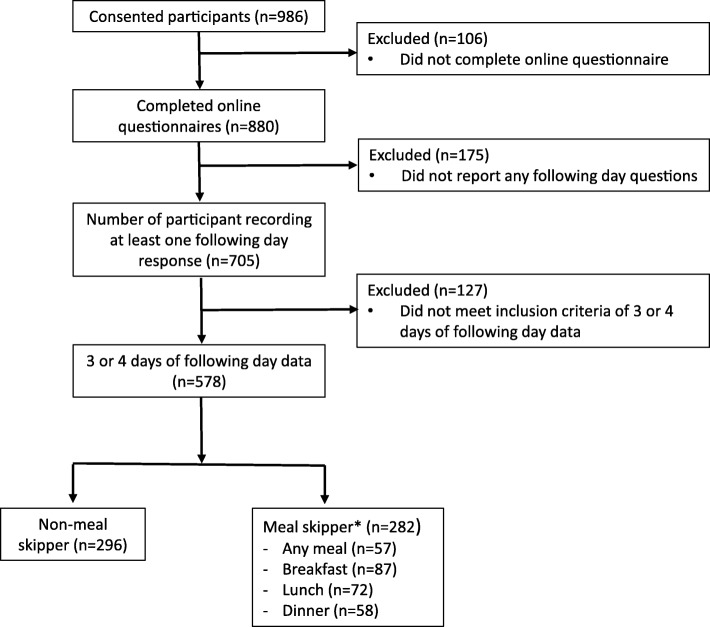
STROBE – nut participant flow diagram (*n* = 986). 273 *Categories not mutually exclusive

Nine hundred and eighty-six participants were recruited into this study. A total of 880 participants (611 female, 269 male) aged 18–30 years (mean age: 24.2 (SD 3.6) years) from Victoria, Australia completed the online questionnaire. Participants’ were excluded from this analysis if they did not complete the online questionnaire (*n* = 106), if they had not completed any following day questions (*n* = 175) or if they had less than three days of following day data (*n* = 127) **(**Fig. 1**).** Three days of dietary data has previously been reported to be sufficient for reporting mean energy intake [18]. A complete case analysis approach was used with regard to excluding participants from analysis.

Five-hundred and seventy-eight participants had three or four days of following day data and were assessed for meal skipping (any meal), breakfast, lunch and dinner skipping. Of this sample, there were 57 meal skippers (any meal) (10%), 87 breakfast skippers (15%), 72 lunch skippers (12%), and 58 dinner skippers (10%). A total of 296 participants did not skip any meals (51%). The majority of the 578 participants held university degrees (56%), were born in Australia (75%), were in a relationship (54%), lived in a suburb ranked with a high SEIFA score (61%), lived outside of the family home (52%), were non-smokers (80%), and met the physical activity guidelines (66%) **(**Table 1**).**

**Table 1:** Socio-demographic and health behaviour characteristics of the young adult participants (*n* = 578)

Characteristic	n (%)^a^
Socio-demographic	
Age (years), Mean ± SD	24.2 ± 3.5
Sex	
Male	139 (24)
Female	439 (76)
Education	
No university degree	235 (40)
University degree	343 (59)
Country of birth	
Australia	437 (76)
Other	141 (24)
Relationship status	
In a relationship	310 (54)
Single	268 (46)
SEIFA	
Low	75 (13)
Medium	151 (26)
High	352 (61)
Living situation	
Living with family	212 (37)
Living alone	66 (11)
Living with flatmates or friends	300 (52)
Health behaviour	
Body mass index (kg/m^2^), Mean ± SD	23.1 ± 4.4
Smoker	
Never	471 (81)
Ex, Occasionally, Regularly smoke	107 (19)
Physical activity	
Do not meet guidelines	208 (34)
Meeting guidelines	370 (66)

^a^Values are n (%) unless otherwise specified

*SEIFA* Socioeconomic Index for Areas (measure of social economic status)

*SD* Standard deviation

### Correlates of meal skipping

Table 2 presents the results of the logistic regression analysis (model 2) that examined associations between potential correlates and meal skipping (any meal), breakfast skipping, lunch skipping and dinner skipping. In model 2, one individual domain variable (education status) remained significantly associated with meal skipping (any meal). Participants with a university degree had lower odds of being a meal skipper (any meal) (54%) compared to those without a university degree (OR = 0.46; 95%CI: 0.22, 0.95; *p* = 0.035).

**Table 2:** Multi-variable adjusted odds ratio and 95% CI of meal skipping behaviours according to correlates from the individual, social-environmental and physical-environmental domains

Variable	Meal skipping (any meal)		Breakfast skipping		Lunch skipping		Dinner skipping	
	OR (95% CI)	P^^^	OR (95% CI)	P^^^	OR (95% CI)	P^^^	OR (95% CI)	P^^^
INDIVIDUAL DOMAIN								
Education								
No University degree	Reference		Reference		Reference		Reference	
University degree	**0.46 (0.22, 0.95)**	**0.035**	–	–	–	–	–	–
Ethnicity								
Australia	Reference		Reference		Reference		Reference	
Other	–	–	1.66 (0.96, 2.86)	0.07	–	–	–	–
Food culture/religion								
Yes	Reference		Reference		Reference		Reference	
No	–	–	–	–	–	–	–	–
Smoking status								
Never	Reference		Reference		Reference		Reference	
Ex, Occasionally, Regularly smoke	1.79 (0.88, 3.65)	0.11	**1.10 (1.15, 3.86)**	**0.016**	**–**	**–**	**–**	**–**
Physical activity								
Not meeting guidelines	Reference		Reference		Reference		Reference	
Meeting guidelines	0.65 (0.35, 1.19)	0.16	–	–	0.61 (0.36, 1.03)	0.07	–	–
Time as a barrier	1.10 (0.96, 1.26)	0.17	**1.12 (1.00, 1.26)**	**0.036**	**1.11 (1.01, 1.23)**	**0.033**	1.11 (0.98, 1.27)	0.11
Self-efficacy to prepare foods	0.98 (0.95, 1.01)	0.20	0.98 (0.96, 1.01)	0.16	–	–	1.00 (0.97, 1.02)	0.86
Food insecurity	–	–	–	–	–	–	1.11 (0.91, 1.36)	0.30
Nutritional knowledge	–	–	–	–	–	–	–	–
Mood	0.99 (0.77, 1.27)	0.93	0.93 (0.75, 1.15)	0.48	–	–	1.16 (0.90, 1.48)	0.25
Hunger	1.20 (0.94, 1.54)	0.14	1.09 (0.89, 1.33)	0.41	1.19 (0.99, 1.41)	0.06	1.00 (0.78, 1.27)	0.30
Weight control	**–**	**–**	–	–	–	–	1.13 (0.89, 1.43)	0.30
Habit	1.05 (0.84, 1.34)	0.66	1.12 (0.93, 1.00)	0.16	–	–	1.10 (0.87, 1.40)	0.42
Taste	–	–	–	–	–	–	–	–
SOCIAL-ENVIRONMENTAL DOMAIN								
Relationship status								
In a relationship	Reference		Reference		Reference			
Single	**–**	**–**	–	–	–	–	–	–
Preference of other people at eating occasion	–	–	–	–	–	–	–	–
PHYSICAL-ENVIRONMENTAL DOMAIN								
SEIFA								
Low	Reference		Reference		Reference			
Medium	–	–	–	–	–	–	–	–
High	–	–	–	–	–	–	–	–
Housing type								
Living with family	Reference		Reference		Reference			
Living alone	–	–	–	–	–	–	–	–
Living with flatmates	–	–	–	–	–	–	–	–
COVARIATES								
Age	–	–	0.92 (0.84, 1.00)	0.06	–	–	0.92 (0.85, 1.00)	0.05
BMI	**1.06 (1.00, 1.12)**	**0.039**	**1.06 (1.00, 1.11)**	**0.032**	1.04 (0.99, 1.10)	0.10	–	–
Sex								
Male	Reference		Reference		Reference			
Female	**0.49 (0.26, 0.93)**	**0.030**	0.68 (0.39, 1.17)	0.17	–	–	–	–

Results presented are the fully adjusted multivariable model, which includes all variables found to be substantially associated with the respective meal skipping behaviour in their Model 1 and age sex and BMI

^*P*-values were calculated using ordinal regression; *P*-Values < 0.05 are bolded

Abbreviations: *CI* confidence intervals, *OR* odds ratio, *BMI* Body Mass Index, *SEIFA* Socioeconomic Index for Areas

Breakfast skipping was associated with two individual domain variables in model 2 (smoking status and time as a barrier). The odds of participants who had previously or currently still smoked cigarettes being a breakfast skipper were 10% higher (OR = 1.10; 95%CI: 1.15, 3.86; *p* = 0.016) compared to those who have never smoked cigarettes. Participants who were time scarce had higher odds (12%) of being a breakfast skipper (OR = 1.12; 95%CI: 1.00, 1.26; *p* = 0.036). Lunch skipping was associated with one individual domain variable (time scarcity). Participants who were time scarce had higher odds (11%) of being a lunch skipper (OR = 1.11; 95% CI: 1.01, 1.23; *p* = 0.033). Dinner skipping was not associated with any variables in model 2.

## Discussion

This study examined the prevalence and correlates of meal skipping (any meal), breakfast skipping, lunch skipping and dinner skipping among young adults using a social-ecological framework. The prevalence of meal skipping varied according to the meal skipping behaviour with 15% of the sample defined as breakfast skippers, 12% as lunch skippers, 10% as dinner skippers and 10% as overall meal skippers (any meal). These meal skipping rates are consistent with those reported in a recent systematic review [8]. Only correlates from the individual domain of the social-ecological framework were associated with meal skipping and correlates varied according to the specific meal skipping behaviours. Not having a university degree was associated with meal skipping (any meal), while time scarcity was associated with both breakfast and lunch skipping, while smoking was associated with breakfast skipping.

In the current study, participants without a university degree had higher odds of being a meal skipper (any meal). While the association between university degree completion and meal skipping behaviours has not previously been investigated, there are well known socioeconomic gradients in dietary intakes [37, 38]. A possible explanation for the associations between meal skipping and education relates to the indirect links between health literacy and education. Health literacy is the capacity to obtain and process basic health information and medical advice, and has been linked with regular meal consumption [39]. Although speculative the link between increased health literacy and meal consumption is thought to be due to individuals being more likely to engage in health promoting behaviours due to their increased understanding of healthy eating messages [39]. Previous literature suggests there is a positive association between health literacy and tertiary education completion [40–42], thus offering an understanding to the association found between university education and total meal skipping seen in this study.

In the current study, time scarcity was positively associated with breakfast and lunch skipping. This is consistent with previous research that has shown that young people’s perception of time is one of the most commonly reported influences on meal skipping [8]. Lack of time or time scarcity refers to the perception of not having enough time to complete the tasks that an individual wants or needs to do in a specific period [43]. Young adults are in a period of transition with multiple priorities and time pressures including further education, employment, and social commitments [44, 45]. These competing demands and time restrictions may place increased demands on the individual, potentially creating a feeling of time scarcity, thus resulting in meal skipping.

Participants who were previous or current smokers were more likely to be breakfast skippers than those who have never smoked cigarettes. These results are consistent with previous studies of both adolescent [46, 47] and adults [47, 48]. Cigarettes contain nicotine, an addictive substance, which has been reported to decrease appetite through the activation of the pro-opiomelanocortin neurons [49]. Due to the effects cigarettes have on feelings of satiety and appetite, many individuals report using smoking as a form of weight control [49]. The addictive nature of nicotine may result in cigarette smoking becoming a habitual behaviour [49]. Previous research on the habits associated with individual eating occasions or meals has shown that the influence of habit is stronger within similar meals than between different meals (due to associations between habits and cues) [50]. For example, the breakfast meal is more likely to be similar in composition to a breakfast meal on a different day of the week when compared to the composition of the dinner meal on the same day [50]. In addition, habits appear to be strongest in the morning due to the generally limited availability of cognitive resources at this time of the day [51]. The association between smoking and breakfast skipping seen in this study could be a result of the nicotine induced addictive habit of replacing a morning breakfast meal with a cigarette.

All correlates reported as significantly associated with meal skipping in this study were from the individual domain of the social-ecological framework. Previous research on meal skipping has identified relationships with correlates from both the social environmental domain and the physical environmental domain of the social-ecological framework [8]. The variable of “being sociable” has been reported in previous research with participants ranking it as an important perceived correlate of meal skipping (any meal) [52]. The current study assessed two social-environment correlates; relationship status and the influence of other people at the eating occasion, with neither variable reported as significant to any meal skipping categories. Physical-environmental correlates such as rural/urban living environments and house type have also been reported to influence meal skipping (any meal) [53, 54]. The current study assessed two physical-environmental correlates (SEIFA and housing type), with neither significantly associated with meal skipping behaviours. This may be explained by the lack of variability within the sample, with 61% of current participants residing in suburbs ranked as high according to the SEIFA definitions. Housing type or living situation was also unevenly distributed across the sample with only 11% residing alone compared to residing with family (37%) or flatmates or friends (52%). This variable has not been tested as a correlate of meal skipping in this format, with previous research only comparing those residing in residential halls compared to non-residential hall living [54]. Further studies with more diverse samples are required to examine these factors further.

The present study has a number of strengths and limitations. Firstly, this study measured meal skipping using a ‘real time’ food diary method with multiple days of dietary data that had been previously evaluated against a comparable valid method [22]. Secondly, this study included a wide range of potential correlates covering multiple domains of the social-ecological framework. Thirdly, online questionnaires were used, which offers the benefits of lower participant and researcher burden whilst lowering administration costs.

As there is currently no consensus on assessment and definitions of meal skipping within the literature [8], the methods and criteria used within this study were based on those from previous studies with a number of refinements. The current study used multiple days of dietary data, and real time data of eating consumption as opposed to recalled consumption and allowed the examination of specific meal skipping behaviours (breakfast, lunch or dinner). The criteria used to classify meal skipping behaviour provided a clearly defined approach for the categorization of specific meal skipping behaviours.

There are a number of limitations of this study. Firstly, the sample was predominantly female, and a large proportion of the sample was of high socio-economic position, and therefore the generalisability of findings is unclear. This is important as previous literature documents sex difference in correlates such as the likeliness to use meal skipping as a weight control technique, with increased rates seen in females compared to males (49 and 18% respectively) [55]. Future research may need to consider alternative recruitment strategies to ensure that a range of participants, varying in sex and social economic position, are recruited to decrease bias associated with the current sample. While Facebook recruitment allows targeted advertisements based on a number of factors such as age, sex and location, recruitment of males and participants from low SES areas was challenging. Therefore, alternative targeted strategies such as collaboration with organisations that males or those from a low SES background are connected to e.g. local sporting teams or events held for young adults may be needed. Related to this is the reduced sample size included in the final analysis, which may introduce bias. Analysis of the original sample, the excluded participants and the final analysis sample suggests that there were no substantial differences with respect to important characteristics such as age, BMI or SEIFA across groups. However, as previously described the analysis sample was predominantly female, and therefore concerns about external validity are still relevant and results should be interpreted in light of this.

Secondly, the cross-sectional design of this study limits inferences of causality. Longitudinal and experimental studies would help to test possible causal pathways between the variables. However, cross-sectional studies may provide important hypothesis generating data in the first instance before moving towards more complex study designs which are relevant when previous literature is limited. Thirdly, this study developed new measures of a number of potential influences on meal skipping (mood, hunger, weight control, habit and taste) as there were no existing measures available Test-retest reliability suggested moderate to fair agreement, which may explain the lack of associations, and therefore further work is required to refine these measures. Further work to develop comprehensive measures of complex constructs such as time scarcity as specific influences over young people’s lifestyle behaviours may improve our understanding of these correlates. Fourthly, education status was measured as a binary variable (university degree versus no university degree). Previous research has shown that any amount of time spent in university education (incomplete university education) can impact health literacy [56], thus future research may warrant examination of this correlate as a continuous variable during analysis.

The present findings have important implications for future eating patterns research. For example, prevalence of meal skipping varies across different eating occasions as do the potential correlates that influence meal skipping. These findings suggest that interventions to reduce specific meal skipping behaviours should be designed taking into consideration the relevant correlates such as time scarcity (breakfast, lunch) and smoking status (breakfast). Interventions should also be developed with consideration of the socio-economic differentials identified in this study, and in other literature [37, 38]. The method of capturing eating occasions and meal skipping used within this study collected data in real time and offers a dietary assessment method with less recall bias than food frequency questions; lower participant and researcher burden compared to 24 h recalls and offers an alternate method for measuring meal skipping in future research.

## Conclusion

The current study found that education, smoking and time scarcity were associated with meal skipping, with variation according to the eating occasion. This study adds to the literature by identifying potential targets for interventions aimed at reducing meal skipping in young adults. Further work is required to examine these factors in longitudinal studies, and to develop further measures of potential influences and correlates specific to meal skipping.

## Additional files

Additional file 1: **Table S1.** List of the measures and responses considered as potential correlates of meal skipping in young adults grouped according to the social-ecological framework. (DOCX 25 kb)

Additional file 2: **Table S2.** STROBE-nut: An extension of the STROBE statement for nutritional epidemiology. (DOCX 28 kb)

## Abbreviations

BMI: Body Mass Index
IPAQ: International Physical Activity Questionnaire
MEALS: Measuring EAting in everyday Life Study
NHANES: National Health and Nutrition Examination Survey
PA: Physical Activity
SD: Standard deviation
SEIFA: Socio-economic Index for Areas
