# Supplementary material for: Examining the correlates of meal skipping in Australian young adults
Source: Nutr J. 2019 Apr 3;18:24. doi: 10.1186/s12937-019-0451-5 (PMC6448264; doi:10.1186/s12937-019-0451-5)
Supplement: Supplementary file 1 — Table S1. List of the measures and responses considered as potential correlates of meal skipping in young adults grouped according to the social-ecological framework. (DOCX 25 kb) [file 12937_2019_451_MOESM1_ESM.docx]

**Table S1. List of the measures and responses considered as potential correlates of meal skipping in young adults grouped according to the social-ecological framework**

|  | **Measure** | **Responses** | **Item type** | **Range** | **Reliability*** |
| --- | --- | --- | --- | --- | --- |
| **Individual influences** | | | | | |
| Education | What is the highest qualification you have completed? | Original response options: *No formal qualifications, Year 10 or equivalent, Year 12 or equivalent, Trade/apprenticeship, Certificate/diploma, University degree, Higher university degree*  Collapsed responses: *University education OR No university education* | Individual item | 1-2 | [38] |
| Ethnicity | Which country were you born in? | Original response options: *Australia, United Kingdom, Italy, Greece, New Zealand, Vietnam, Other*  Collapsed responses: *Australia OR Other* | Individual item | 1-2 | [48] |
| Culture/religion | Do you avoid any particular foods because of cultural, religious or ethical reasons? | *Yes/No* | Individual item | 1-2 | Adapted from [39]  Ƙ=0.73^2^ |
| Smoking status | Which of the following best describes your current smoking status? | Original response options: *Never smoked, Used to smoke, Smoke occasionally, Smoke regularly*  Collapsed responses: *Never OR*  *Ex, Occasionally, Regularly smoke* | Individual item | 1-2 | [38] |
| Physical activity | International Physical Activity Questionnaire (IPAC) - Short form | *Total minutes/week of vigorous intensity PA,*  *Total minutes/week of moderate intensity PA*  Collapsed responses: *Meet PA guidelines OR Do not meet PA guidelines* | Individual item | 1-2 | α= 0.51–0.64  r =0.58^1^  [40] |
| Time scarcity | 4 items  A) I am too busy to eat healthy foods  B) I am too rushed in the morning to eat a healthy breakfast  C) Eating healthy meals just takes too much time  D) I don’t have time to think about health eating | Original response options: *Strongly disagree, Disagree, Agree, Strongly agree*  *(Scored 1-4)*  Sum to form total score. | Scale | 4-16 | Project EAT-III  α = 0.79^3^  r = 0.62^1^  [42] |
| Self-efficacy | 17 item scale was used to assess personal self-efficacy related to health-related diet behaviours | Original response options: *Not at all confident, Slightly confident, Moderately confident, Very confident, Extremely confident*  *(Scored 1-5)*  Sum to form total score. | Scale | 17-85 | Adapted from Sallis. [43] |
| Food insecurity | 5 items (Last 12 months)  A) The food that I/we bought just didn’t last and I/we didn’t have enough money to get more  B) I/we couldn’t afford to eat balanced meals  C) Did you or other adults in your household ever cut the size of your meals or skip meals because there was not enough money for food?  D) Did you ever eat less than you felt you should because there was not enough money to buy food?  E) Were you ever hungry but didn’t eat because you couldn’t afford enough food? | Original response options: *Often true, Sometimes true, Never, Don’t know/don’t want to answer*  *OR*  *Yes-almost every month, Yes-some months but not every month, Yes-only 1 or 2 months, No*  Often true, sometimes true, Yes, almost every month, yes are all marked as affirmatives =1  Sum to form total score. | Scale | 0-5 | Guide to measuring household food security  Sensitivity 92% Specificity 99.4%  [44] |
| Nutritional knowledge | 51 item scale General Nutrition Knowledge Questionnaire to assess the relationship between knowledge and dietary behaviour | Correct items get a score of 1.  Sum to form total score. | Scale | 0-51 | α= 0.53-0.92^3^  r= 0.87^1^  [45] |
| Mood | How likely are the following factors going to result in SKIPPING a main meal? | *1= Not likely, 2, 3, 4, 5 = Highly likely* | Scale | 1-5 | Ƙ=0.41^2^ |
| Hunger | How likely are the following factors going to result in SKIPPING a main meal? | *1= Not likely, 2, 3, 4, 5 = Highly likely* | Scale | 1-5 | Ƙ=0.44^2^ |
| Weight control | How likely are the following factors going to result in SKIPPING a main meal? | *1= Not likely, 2, 3, 4, 5 = Highly likely* | Scale | 1-5 | Ƙ=0.39^2^ |
| Habit | How likely are the following factors going to result in SKIPPING a main meal? | *1= Not likely, 2, 3, 4, 5 = Highly likely* | Scale | 1-5 | Ƙ=0.42^2^ |
| Taste | How likely are the following factors going to result in SKIPPING a main meal? | *1= Not likely, 2, 3, 4, 5 = Highly likely* | Scale | 1-5 | Ƙ=0.39^2^ |
| **Social-environmental influences** | | | | | |
| Relationship status | Which of the following best describes your relationship status? | Original responses: *Married, De facto relationship, Committed dating relationship or engaged, Single, Separated, Widowed*  Collapsed responses: *In a relationship OR Not in a relationship* | Individual item | 1-3 | [48] |
| Preference of other people at eating occasion | How likely are the following factors going to result in SKIPPING a main meal? | *1= Not likely, 2, 3, 4, 5 = Highly likely* | Scale | 1-5 | Ƙ=0.32^2^ |
| **Physical-environment influences** | | | | | |
| Area level Social-economic position | What is your current address? | *SEIFA*  *1-4= Low*  *5-7 = Medium*  *8-10 = High*  Collapsed responses: *Low OR Medium OR High* | Individual item | 1-3 | [46] |
| Housing type | Which of the following best describes your current housing or living situation? | Original responses: *Living with parents/family, Living by myself, Living with partner/spouse, Living with flatmates/friends, Living with children, Other*  Collapsed responses: *Living with family OR Living alone OR Living with flatmates/friends* | Individual item | 1-3 | [48] |
| **Covariates** | | | | | |
| Sex | What gender do you identify as? | *Male, Female* | Individual item | 1-2 | [48] |
| Age | What is your date of birth? | *dd/mm/yyyy* | Individual item | Continuous | [48] |
| Self-reported weight | How much do you currently weight, without clothes of shoes? | *Kg* | Individual item | Continuous | [47] |
| Self-reported height | How tall are you without shoes? | *cm* | Individual item | Continuous | [47] |

PA = Physical activity, PA guidelines = (>150 mins of moderate or >75 mins of vigorous activity per week) [41], SEIFA = Socioeconomic Index for Areas

All items were measured in the online survey

* Where possible reference of validation study is provided for each measure. For measures that were created specifically for this analysis test-retest weighted kappa is presented.

^1^r = test-retest Pearson correlation

^2^Ƙ =weighted kappa

^3^α= alpha
